# Novel Hump Measurement System With a 3D Camera for Early Diagnosis of Patients With Adolescent Idiopathic Scoliosis: A Study of Accuracy and Reliability

**DOI:** 10.7759/cureus.8229

**Published:** 2020-05-21

**Authors:** Tatsuya Sato, Ikuho Yonezawa, Toshinari Akimoto, Terada Nobuyuki, Yukitoshi Shimamura, Osamu Muto, Teppei Suzuki, Rei Momomura, Koki Uno, Ken Yamazaki, Kenta Fujiwara, Akiko Misawa, Kazuo Kaneko

**Affiliations:** 1 Orthopaedic Surgery, Juntendo University School of Medicine, Bunkyo-ku, JPN; 2 Orthopaedics, Sangubashi Spine Surgery Hospital, Tokyo, JPN; 3 Robotics, Nippon Institute of Technology, Minami Saitama-gun, JPN; 4 Science and Engineering: Biomedical Engineering, Toyo University, Bunkyo-ku, JPN; 5 Orthopaedic Surgery, Juntendo University, Bunkyo-ku, JPN; 6 Orthopaedic Surgery, Kobe Medical Center, Kobe, JPN; 7 Orthopaedic Surgery, Juntendo Unversity, Bunkyo-ku, JPN; 8 Iwate Spinal Scoliosis Center, Tochinai Daini Hospital, Takizawa, JPN; 9 Orthopaedic Surgery, Osaka Medical College, Takatsuki, JPN; 10 Orthopaedic Surgery, Prefectural Center on Development and Disability, Akita, JPN

**Keywords:** three-dimensional camera, scoliosis screening, moiré topography, digital moiré

## Abstract

Background

Adolescent idiopathic scoliosis (AIS) is a potentially progressive deformity, and early detection is crucial for timely intervention. However, the methods and criteria justifying screening for pediatric scoliosis remain controversial. We have, therefore, independently developed a Digital Moiré (DM) as a tool for scoliosis screening. The purpose of this study was to assess the usefulness of DM for scoliosis screening.

Methods

From March 2016 to March 2017, 126 patients (18 boys, 108 girls, mean age: 13.2 ± 2.2 years) with AIS underwent radiographic imaging of their whole spine. We tested the accuracy and reliability of DM by categorizing the examination results as Class 0 (no abnormality), Class 1 (return visit in one year), and Class 2 (further examination needed) and determined the distribution of the population by Cobb angle. The intra/inter-rater reliability and receiver operating characteristic (ROC) analyses were used to categorize the patients with positive findings into Class 1 or 2.

Results

Regarding the population distribution per Cobb angle in each of the distributions, 11 patients (8.7%) were Class 0, of which nine and two patients had Cobb angle ≤ 10 ° and > 10 °, respectively. There were 20 (15.9% ) Class 1 cases, of which 17 and three had Cobb angle ≤ 10 ° and > 10 °, respectively. Of the 95 (75.4%) Class 2 cases, five and 90 had a Cobb angle of ≤ 10 ° and > 10 °, respectively. The receiver operating characteristic (ROC) analysis of patients with positive findings showed that the area under the curve (AUC), sensitivity, specificity, and false-positive rate were 0.76, 0.98, 0.53, and 0.47, respectively, when predicting Cobb angle > 10°. Intra-rater and inter-rater reliability were 0.73 and 0.70, respectively.

Conclusions

This study demonstrated the usefulness of DM for determining whether a child with AIS requires a follow-up observation such as radiograph. Our findings suggest that the novel DM shows high accuracy and reliability for scoliosis screening.

## Introduction

Adolescent idiopathic scoliosis (AIS) is a lateral spinal curve > 10° found among children from 10-18 years with no prior history of scoliosis [1]. The treatment that should be considered for AIS includes bracing if the curve is ≥ 25° or surgery if the curve is ≥ 45° [2]. Early detection of this potentially progressive deformity is crucial for early intervention and treatment. Several methods are available for school scoliosis screening (SSS) aimed at early detection. Radiographic image-based diagnosis is reliable; however, children should not be exposed to radiation for the purpose of screening [3-4]. Adam’s forward bend test using a scoliometer is widely used; however, it also has limitations, such as low correlation to the Cobb angle or need for the rater to perform the forward bend or measure data manually and within a limited time [5-6]. Conventional Moiré topography (MT) is a method of evaluating the shape of the trunk based on the external body contour [7]. However, a limitation of MT is the high rate of false positives at 35%-67%; yet, it continues to be used in certain regions in Japan for examination [8-9]. The production of cameras for MT (FM40SC, made by Fujifilm Medical, Japan) was discontinued 10 years ago.

In 2013, we began to develop a new SSS tool using 3D cameras that could replace MT [10]. We have since completed a surface topography tool, Digital Moiré (DM), with a built-in 3D camera. The purpose of the present study was to determine the accuracy and reliability of our newly developed DM for the purposes of AIS screening.

## Materials and methods

**Figure 1:**
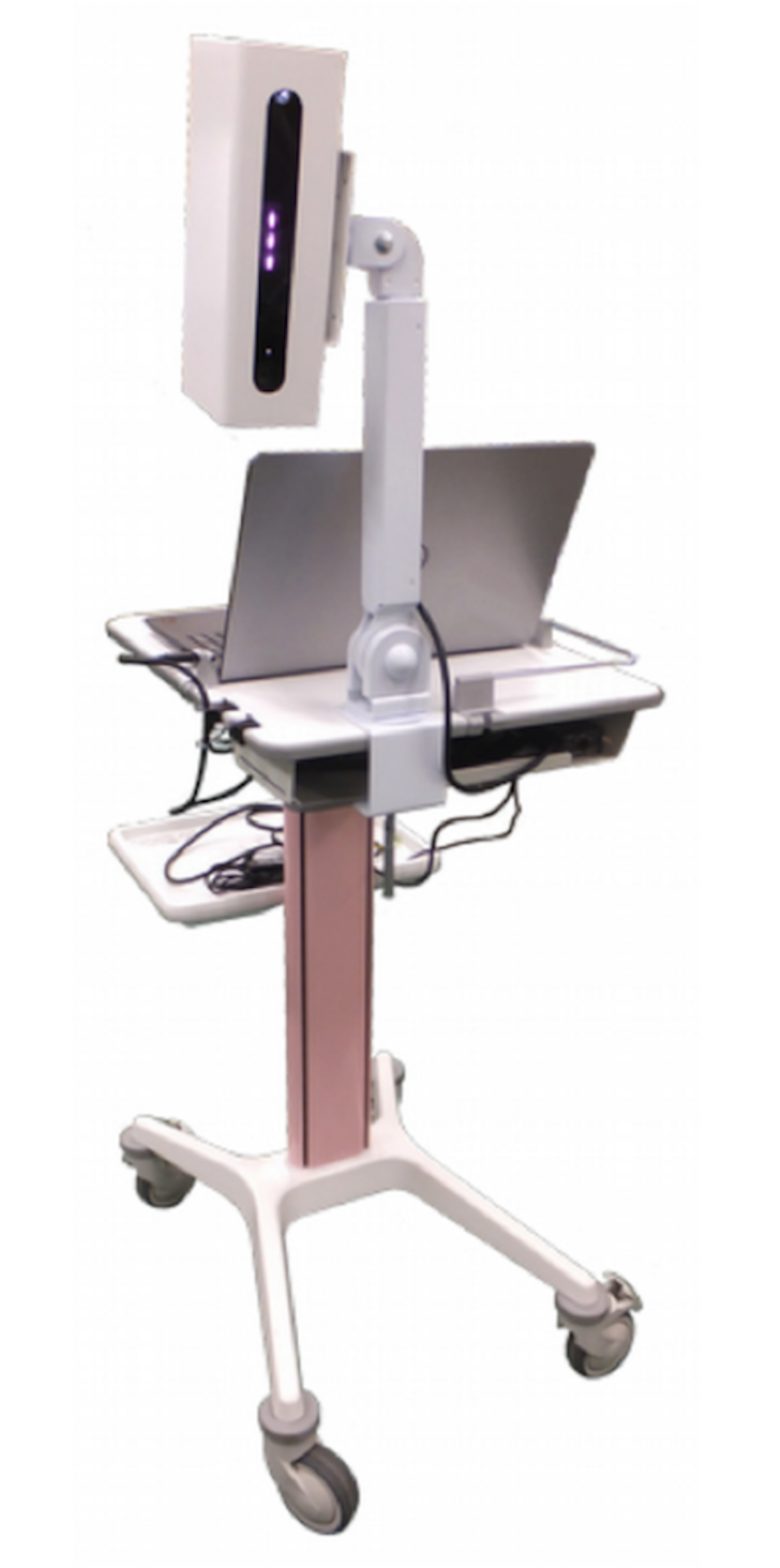
Hump measurement system with a built-in 3D camera View of Digital Moiré

**Figure 2:**
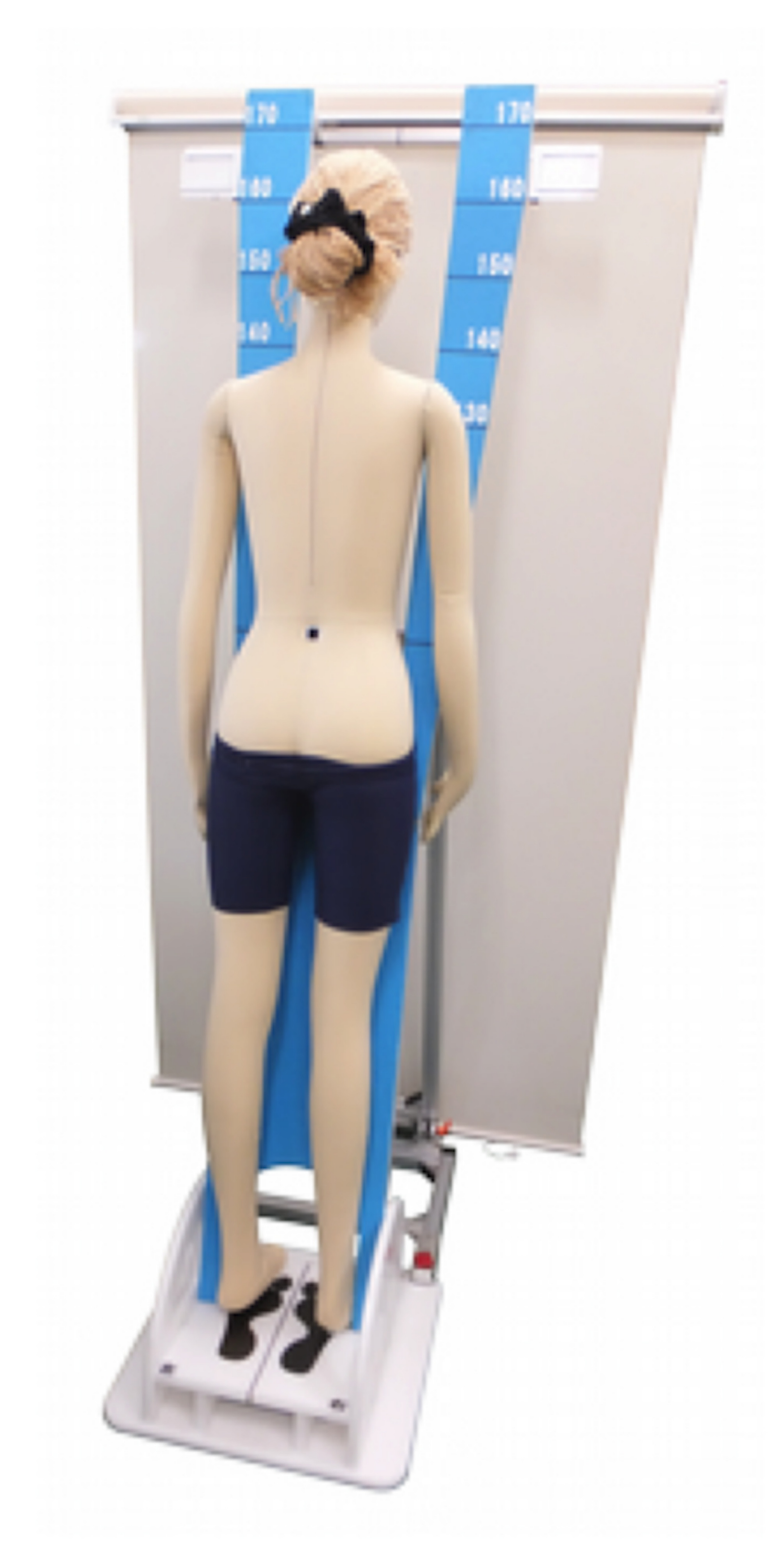
Stand for Digital Moiré imaging Employing a similar stand to the one for a conventional Moiré Topography (MT) examination

From March 2016 to March 2017, 126 patients (18 boys, 108 girls, mean age: 13.2 ± 2.2 years) with AIS underwent X-ray imaging of their whole spine. The DM is configured using the 3D camera (Kinect for Windows: Microsoft Corporation, Redmond, Washington) and a personal computer (PC) for analysis (Figures 1-2).

**Figure 3:**
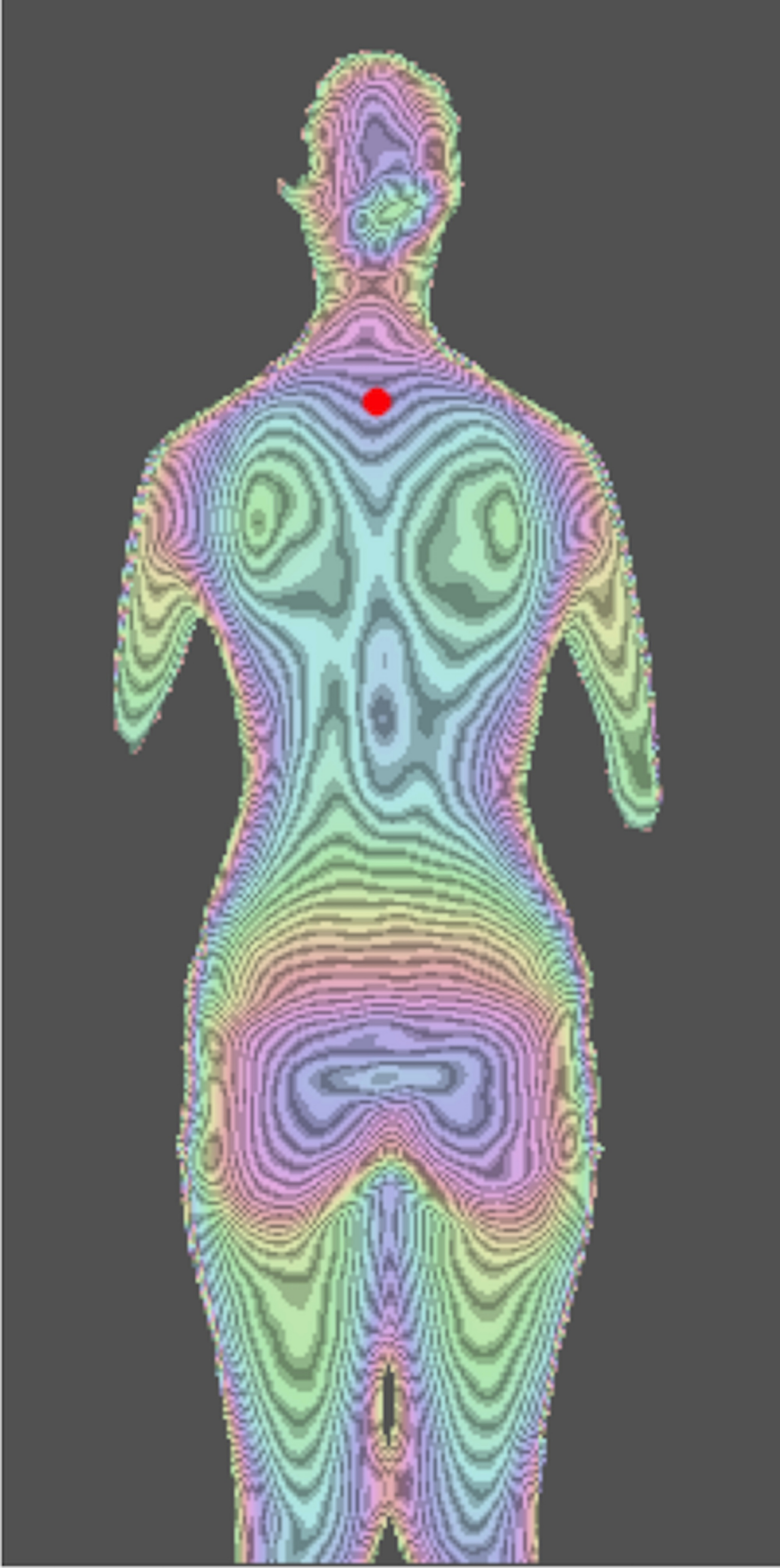
Digital moiré image of the subject The larger the deviation, the deeper the color

**Figure 4:**
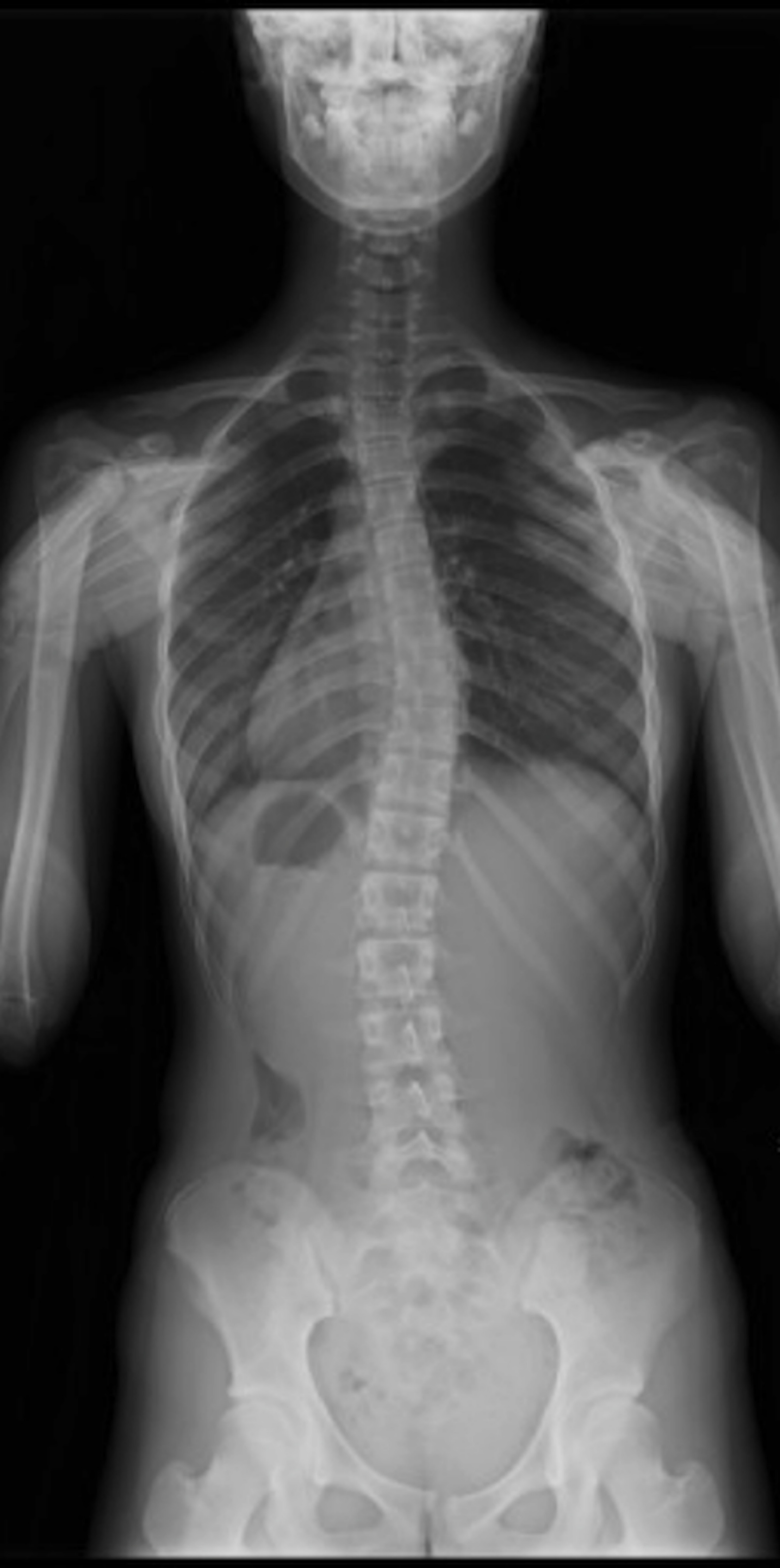
Standing posteroanterior radiograph (whole spine) of the same subject Cobb angle 16° for T5-T11

Imaging was done as with MT, with the subject standing while leaning approximately 10° forward with both feet aligned on a posture holder stand. The 3D camera captures images from about 1.2 m (variable from 0.5 to 4.0 m) behind the subject. The 3D camera is based on laser-pattern projections, where the infrared light projected from the 3D camera was subsequently captured by the analysis PC's unique image data, from which the PC constructs an image similar to that of the MT (Figure 3) with similar information as with a standing posteroanterior radiograph of the whole spine (Figure 4).

The consecutive topographical contour lines were at 5-mm intervals, and assessments could be made using the MT assessment method. Image information was obtained at a maximum rate of 30 frames/second, and the rater could finely adjust the subject’s body position during imaging, similar to MT, making it possible to have the subjects in a uniform posture. Results were assessed in the same way by two different spinal surgeons, using a classification system similar to that while assessing MT-Class 0 - no abnormality, Class 1 - return visit in 1 year, and Class 2 - further examination needed.

The population density per Cobb angle in each assessment was aggregated to assess the accuracy of the DM. The receiver operating characteristic (ROC) analysis of patients with positive findings for predicting Cobb angles > 10°, 15°, or 20° was used to assess the accuracy of the DM. The intra/inter-rater reliability for patients with positive findings was also analyzed.

For the statistical analyses, DM performance was assessed using the area under the ROC curve (AUC). AUCs were categorized as follows: no discrimination (AUC = 0.50); acceptable discrimination (0.7 ≤ AUC < 0.8); excellent discrimination (0.8 ≤ AUC < 0.9); and outstanding discrimination (AUC ≥ 0.9) [11]. Intra/inter-rater reliability concerning patients with findings was assessed using the intraclass correlation coefficients (ICC). The extent of agreement according to Fleiss’ κ coefficient was determined to be “poor” for κ ≤ 0.40, “moderate” for 0.40 < κ ≤ 0.60, “substantial” for 0.60 < κ ≤ 0.80, and “almost perfect” for 0.80 < κ [12]. The level of significance was defined as < 5%. All analyses were performed using the IBM SPSS Statistics 21 software package (IBM Japan, Tokyo).

## Results

**Figure 5:**
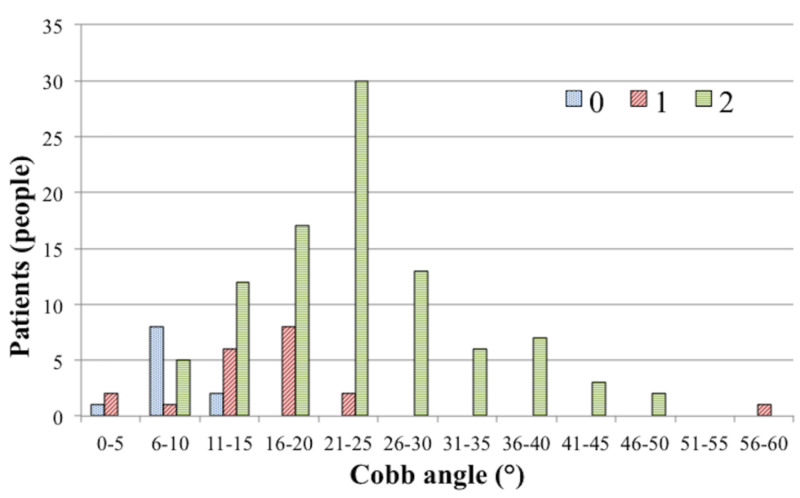
The population per Cobb angle in each assessment

Among 11 patients with Class 0, the Cobb angle in one, eight, and two patients was 0°-5°, 6°-10°, and 11°-15°, respectively. Among 20 patients with Class 1, the Cobb angle in two, one, six, eight, two, and one patients was 0°-5°, 6°-10°, 11°-15°, 16°-20°, 21°-25°, 56°-60°, respectively. Among the 95 patients with Class 2, the Cobb angle in five, 12, 17, 30, 13, six, seven, three, and two patients was 6°-10°, 11°-15°, 16°-20°, 21°-25°, 26°-30°, 31°-35°, 36°-40°, 41°-45°, and 51°-55°, respectively (Figure 5).

In the ROC analysis for patients with findings, when predicting a Cobb angle > 10°, the AUC was 0.76, sensitivity was 0.98, specificity was 0.53, the false-positive rate was 0.47, false-negative rate was 0.02, predictive value was 0.93, negative predictive value was 0.82, accuracy was 0.97, positive likelihood ratio was 2.09, and negative likelihood ratio was 0.04. The AUC was determined to have acceptable discrimination ability. Results based on Cobb angles of >10°, >15°, and >25° are shown in Table 1.

**Table 1:** ROC analysis with Cobb angle ROC = receiver operating characteristic, AUC = area under the curve, FPR = false-positive value, FPR = false-negative value, PPV = positive predictive value, NPV = negative predictive value, PLR = positive likelihood ratio, NLR = negative likelihood ratio.

Cobb angle	AUC	Sensitivity	Specificity	FPR	FNR	PPV	NPV	PLR	NLR
> 10	0.76	98.2	52.9	47.1	1.8	93	81.8	2.09	0.04
> 15	0.67	100	34.4	65.6	0	77.3	100	1.52	0
> 25	0.56	100	11.7	88.3	0	27.8	100	1.13	0

The intra-rater agreement rate for those with positive findings categorized as Class 1 or 2 was substantial (κ coefficient 0.73, p < 0.001), and the inter-rater agreement rate were substantial (κ coefficient 0.70, p < 0.001).

## Discussion

SSS preferably involves a standardized method that can be objectively assessed; however, the usefulness of SSS itself remains a matter of debate [13]. The methods of SSS are inconsistent and vary, including visual inspection and palpation, Adam’s forward bend test only, the combined use of Adam’s forward bend test and a scoliometer, or MT [14]. Some reports recommend the scoliometer; however, there are issues, such as its weak correlation to the Cobb angle or the need for the rater to perform the forward bend or manual data measurement within a limited time [5-6,15]. MT also shows a high rate of false positives, at 35%-67% [8-9]. However, in Japan, it has continued to be widely used since the 1980s in some regions to this day as an objective method of screening [8]. Furthermore, MT cameras are no longer in production, and no successor machines exist. Therefore, we developed the DM to build an SSS tool to replace MT.

The present study showed that not even a single child with Class 0 had a Cobb angle > 15° and that examination had a sensitivity of 98.2% and a false-positive rate of 47.1% when the cut-off value was set to a Cobb angle of 10°. The sensitivity was adequately high, and the false-positive rate was equivalent to that of the MT in Japan [8-9]. Therefore, this suggests that it is sufficiently accurate as a screening method. The high intra-rater and inter-rater reproducibility also suggest that it is a comparably reliable screening method.

Many studies have constructed a quantitative screening system for scoliosis. Watanabe et al. attempted to predict scoliosis from the software that uses artificial intelligence to predict spinal alignment by looking at MT images [16]. Komeili A et al. stated that surface topography without markers could be employed to reduce the number of radiographic examinations [17]. Sudo et al. reported developing a new screening system for scoliosis by using Adam’s forward bend test with reportedly good results [6].

Compared to other scoliosis screening systems, our DM has five noteworthy features. First, infrared light is used, and there is no exposure to radiation. Second, the physical design is small and easy to relocate and install. Third, because the assessment is done in standing posture, the obtained measurement results are stable, regardless of the extent of forward-bending. Fourth, posture can be finely adjusted during the examination. Fifth, the imaging method and results assessment resemble MT because the rater gains an understanding of the images without resistance.

There are three significant limitations to the present study. First, because the subjects were outpatient examinees, there might have been a selection bias leading to results that might be different from SSS as a primary screening method. Second, the scoliosis curve type was not considered, and third, objective results were not obtained as the digitization of the body surface shape was used.

Constructing an SSS for diagnosing scoliosis without radiation exposure is an important task. In Japan, where MT continues to be the widespread form of SSS, the discontinuation of the production of the MT cameras might result in a potential for DM as a tool for SSS [8]. Currently, several Japanese regions are already introducing DM screening, and further studies based on those results are warranted to further elucidate its efficacy.

## Conclusions

Because of the discontinuation of the production of MT examination machines, we have developed a novel DM as a hump measurement system with a built-in 3D camera. DM has sufficient accuracy and reproducibility and has demonstrated excellent screening capability for determining whether or not a child with AIS requires further follow-up such as radiography. Our results suggest that our DM examination is useful as a new method for the screening of scoliosis with sufficient accuracy and reliability to replace MT.
